# Draft Genome Sequence of Bioactive-Compound-Producing Cyanobacterium Tolypothrix campylonemoides Strain VB511288

**DOI:** 10.1128/genomeA.00226-15

**Published:** 2015-04-02

**Authors:** Subhadeep Das, Deeksha Singh, Madhavi Madduluri, Mathu Malar Chandrababunaidu, Akash Gupta, Siba Prasad Adhikary, Sucheta Tripathy

**Affiliations:** a Structural Biology and Bioinformatics Division, CSIR, Indian Institute of Chemical Biology, Kolkata, West Bengal, India; b Fakir Mohan University, Vyasa Vihar, Nuapadhi, Balasore, Odisha, India

## Abstract

We report here the draft genome sequence of Tolypothrix campylonemoides VB511288, isolated from building facades in Santiniketan, India. The members of this genus produce several compounds of commercial importance. The draft assembly is 10,627,177 bases in 135 scaffolds, and it contains 7,886 protein-coding genes, 994 pseudogenes, 18 rRNA genes, and 76 tRNA genes.

## GENOME ANNOUNCEMENT

Tolypothrix is a genus of aerophytic heterocystous cyanobacteria and belongs to the order *Nostocales* (1). These filamentous organisms occur in fasciculated colonies in which the heterocystic basal parts and free apical ends are mostly falsely branched. Tolypothrix spp. are commonly found in floating tufts or submerged in fresh torpid water, attached to plants or rocks, or are sometimes reported from mineral springs (http://www.cyanodb.cz). Organisms belonging to the genus Tolypothrix produce several metabolites having commercial importance. Some Tolypothrix species can be directly used as biofertilizers (2) or can be a rich source of fatty acids (3). Many strains of Tolypothrix are known to produce anti-inflammatory (4), antibacterial (5), and antifungal compounds (6).

Tolypothrix campylonemoides VB511288 was collected as greenish biofilms from the exterior of lime-washed building facades in Santiniketan, eastern India, and was grown in BG11 agar medium at room temperature (~28°C) under 16-h light/8-h dark conditions with periodic (once a day) shaking. DNA isolation and purification were done using the UniFlex bacterial DNA isolation kit (Genei). A total of 4 µg of genomic DNA was provided for sequencing, of which 1 µg was used for shotgun sequencing and 3 µg for mate-pair sequencing.

Genome sequencing was carried out using an Illumina HiSeq platform. Two different libraries were constructed, a paired-end library with an insert size of 300 bp and a mate-pair library with an insert size 3 kb in opposite orientations. Sequencing was done, generating 151-bp read fragments for the paired-end library, at 103× coverage (~10,493,948 reads), and 101 bp fragments for the mate-pair library, at 72.4× coverage (~5,159,490 reads). The raw reads from both libraries were filtered and preprocessed using the SGA (7) and TagDust (8) algorithms to remove poor-quality reads. The resulting 10,417,561 high-quality paired-end reads and 5,159,490 mate-pair reads were assembled using the AllPaths-LG (9) assembler. The assembly generated 135 scaffolds and a genome size of 10,627,177 bp, with an *N*_50_ of 1,097,626 bp. The largest and smallest scaffolds are 1,728,859 bp and 10,190 bp long, respectively, and the total G+C content is 48%.

The genome was annotated using the NCBI PGAAP (http://www.ncbi.nlm.nih.gov/genome/annotation_prok/). The total number of predicted genes was 8,794, out of which 994 are pseudogenes. There were 14 clustered regularly interspaced short palindromic repeat (CRISPR) arrays, 1 noncoding RNA (ncRNA) gene, 76 tRNA genes, and 132 frameshifted genes predicted from this assembly. Several antibiotic resistance genes (e.g., vancomycin and β-lactamase), genes associated with the degradation of toxic compounds (e.g., nitrotoluene degradation), and some nonribosomal proteins with antifungal activities (e.g., those in the fengycin family) were annotated in this assembly. Several proteins, such as arsenic transporters, were found in the genome, which need to be further investigated for their role in bioremediation.

### Nucleotide sequence accession number.

The T. campylonemoides VB511288 genome sequence and annotation data have been deposited in GenBank under the accession no. JXCB00000000.
